# Synchrotron Microtomography Reveals the Fine Three-Dimensional Porosity of Composite Polysaccharide Aerogels

**DOI:** 10.3390/ma10080871

**Published:** 2017-07-28

**Authors:** Abdul Ghafar, Kirsti Parikka, David Haberthür, Maija Tenkanen, Kirsi S. Mikkonen, Jussi-Petteri Suuronen

**Affiliations:** 1 Department of Food and Environmental Sciences, P.O. Box 66 (Agnes Sjöbergin katu 2), University of Helsinki, FI-0014 Helsinki, Finland; kirsti.parikka@helsinki.fi (K.P.); maija.tenkanen@helsinki.fi (M.T.); kirsi.s.mikkonen@helsinki.fi (K.S.M.); 2 X-ray Tomography Group, Swiss Light Source, Paul Scherrer Institute, 5232 Villigen, Switzerland; david.haberthuer@ana.unibe.ch; 3 ESRF—The European Synchrotron, CS40220, Grenoble CEDEX 9, 38043 Grenoble, France; jussi-petteri.suuronen@esrf.fr

**Keywords:** polysaccharide, nanofibrillated cellulose, ice-templating, synchrotron microtomography, image analysis

## Abstract

This study investigates the impact of ice-templating conditions on the morphological features of composite polysaccharide aerogels in relation to their mechanical behavior and aims to get a better insight into the parameters governing these properties. We have prepared polysaccharide aerogels of guar galactomannan (GM) and tamarind seed xyloglucan (XG) by enzymatic oxidation with galactose oxidase (GaO) to form hydrogels, followed by conventional and unidirectional ice-templating (freezing) methods and lyophilization to form aerogels. Composite polysaccharide aerogels were prepared by incorporating nanofibrillated cellulose (NFC) into polysaccharide solutions prior to enzymatic oxidation and gel formation; such a cross linking technique enabled the homogeneous distribution of the NFC reinforcement into the gel matrix. We conducted phase-enhanced synchrotron X-ray microtomography (XMT) scans and visualized the internal microstructure of the aerogels in three-dimensional (3D) space. Volume-weighted pore-size and pore-wall thickness distributions were quantitatively measured and correlated to the aerogels’ mechanical properties regarding ice-templating conditions. Pore-size distribution and orientation depended on the ice-templating methods and the NFC reinforcement that significantly determined the mechanical and shape-recovery behavior of the aerogels. The results obtained will guide the design of the microporous structure of polysaccharide aerogels with optimal morphology and mechanical behavior for life-sciences applications.

## 1. Introduction

Polysaccharides are abundant in nature, and their transformation into a highly porous and low-density solid material called aerogel has attracted more attention over the past few years due to their excellent intrinsic properties [1,2,3] for life-sciences applications [4,5]. The term ‘aerogel’ was firstly used for solid foams prepared from silica using a supercritical CO_2_ drying technique, which allows the retention of the gel network in a dry state [6]. Later, Quignard et al. described the aerogel as “a gel which has been dried retaining the dispersion of the wet state’’ [3]. However, the highly porous and low-density solid materials are called either aerogels from supercritically dried gels [6], cryogels from freeze-dried/lyophilized gels [7], or foams [8]. Currently, the term ‘aerogel’ is becoming generally accepted for low-density and highly porous solid structures, regardless of whether they are lyophilized or supercritically dried [9,10]. The first step in aerogel preparation is the formation of a liquid gel such as a hydrogel. Hydrogels are 3D networks of cross-linked polymers capable of imbibing a large amount of water. Hydrogels are transformed to aerogels by extraction of the liquid (drying), using techniques capable of maintaining the hydrogel’s 3D structure in the dry state. The unique properties of aerogels greatly depend on the drying method used because variations in the microporous structure have a direct impact on the performance of the resulting material [3,11]. We successfully presented an enzymatic oxidation technique using galactose oxidase (GaO) for crosslinking polysaccharides (guar galactomannan (GM) and tamarind seed xyloglucan (XG)) [12], resulting in the formation of hydrogels and subsequently in lyophilization or supercritical CO_2_ drying to form aerogels [13,14]. The catalytic activity of GaO was not hindered with the addition of nanofibrillated cellulose (NFC) as a reinforcing agent in polysaccharide composite aerogels [14,15]. 

Lyophilization (freeze-drying) is a simple and, compared to supercritical CO_2_ drying [1], more versatile method to produce polysaccharide-based aerogels, and it permits more control over the macroporosity of the aerogel, enabling the gel to be tailored to specific applications [7,9]. Imitating nature’s highly sophisticated structural organization in relation to optimal function [16], researchers have used the ice-templating method with unidirectional freezing to develop aerogels with tailored morphological features such as honeycomb or lamellar structures [17,18,19,20,21]. Due to its simplicity, the ice-templating process with unidirectional freezing has been applied to various materials [18,19,22,23] to obtain aerogels that show optimal mechanical behavior [24,25,26]. However, the homogenous distribution of an aligned microporous structure throughout the monolithic aerogel is hard to achieve [18]. The pore size of the aerogels is considered to be a critical parameter in biomedical applications as a scaffold for soft-tissue regeneration [5,27] because the minimum pore size of the scaffold must exceed the cell size; otherwise, penetration of the cells into the scaffold is compromised. The interconnectivity of the pores is a valuable asset of the aerogel that facilitates the movement of nutrients and oxygen to the growing cells, as well as the transportation of metabolic products from the cells. Pore sizes in the range of 100 μm to 500  μm were considered optimal for tissue regeneration [28]. The versatility of the ice-templating method followed by lyophilization provides a great freedom for developing porous materials with optimal morphology [29].

Currently deployed probing techniques for the structural characterization of aerogels such as scanning electron microscopy (SEM) and focused ion beam SEM (FIB-SEM) are limited to surface- level characterization. It is still difficult to characterize the actual representation of the 3D structure of the aerogels, the connectivity of the pores, the pore-size distribution, and the interface of the reinforcement with polymers within the aerogel matrix. Recent advancements in experimental techniques enable us to visualize and quantify the 3D morphology of the materials at the micron and sub-micron levels. Synchrotron X-ray microtomography (XMT) is an advanced and non-invasive analytical tool to illustrate the detailed internal structure of a material such as an aerogel. The technique has been used across several disciplines, including physics, materials science, medicine, and powder technology [30]. The porous structure of NFC-silica hybrid aerogels and the distribution of NFC in the aerogel matrix were recently successfully observed using XMT due to the highly different attenuations of X-rays by NFC and silica [31]. The 3D porous structure of NFC-xylan composite foam was viewed using microcomputed tomography [32] that provided less detailed illustrations than synchrotron XMT, and we have earlier visualized a single polysaccharide-based aerogel sample with XMT [13]. The previous work raised interest in a systematic morphological comparison of ice-templated biocomposite aerogels, including the determination of quantitative parameters such as pore size and pore wall thickness distribution. 

In this work, enzymatic crosslinking was used to obtain GM and XG hydrogels and NFC-reinforced, completely bio-based composite hydrogels. Both conventional and unidirectional freezing techniques were used as ice-templating methods for hydrogels that were subsequently lyophilized to obtain aerogels. The aim of this study was to see, using synchrotron XMT, the influence of processing conditions (ice-templating methods) and reinforcement regarding polysaccharide types on the qualitative and quantitative morphological features of the aerogels. The qualitative and quantitative morphological features of the aerogels were correlated with the mechanical performance of the aerogels. This information will contribute toward more understanding of the structure-function properties of the biocomposite aerogels and how the XMT technique applies to the characterization of lightweight and highly porous materials.

## 2. Results and Discussion

The resolution of XMT images depends on the dimensions of the aerogel samples. Cutting polysaccharide aerogel samples into small sizes (e.g., <1 mm width × length) for an XMT scan is difficult due to the elastic interconnected structure of the polysaccharide aerogels, which was experienced in our earlier study [13]. Therefore, the samples presented in this paper were prepared and characterized inside the small polyether-ether-ketone (PEEK) capillaries (Section 3.2), unless otherwise mentioned, to avoid any structural damage at the sample-preparation stage. The PEEK capillary did not hinder the XMT signals. The results from XMT were used to visualize the internal porous structure of the aerogels in 3D space. Furthermore, the results obtained from the quantitative analyses provided essential information for characterizing and examining the correlation of aerogels’ microstructures to the polysaccharide types, processing parameters, and mechanical performance.

### 2.1. 3D Structural Visualization

**Figure 1:**
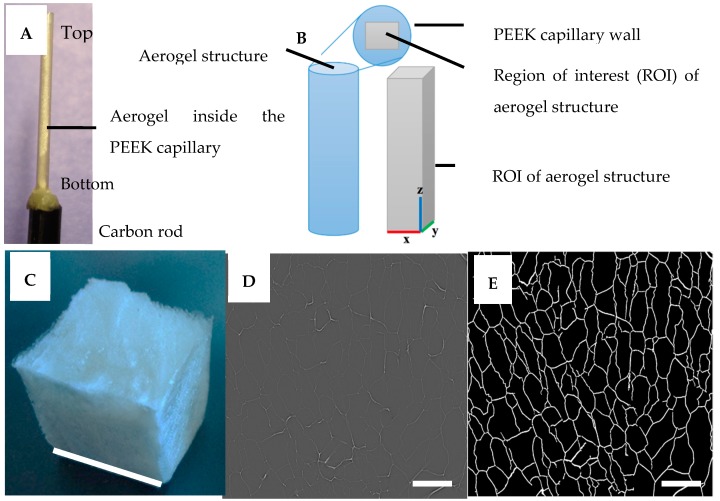
(**A**) Formation of aerogels inside the polyether-ether-ketone (PEEK) capillary (Diameter: 0.8175 mm); (**B**) Graphical representation of the selected region of interest (ROI) of the aerogel structure to omit the PEEK capillary wall; (**C**) Cubical aerogel; (**D**) Tomographic image from the Tomographic Microscopy and Coherent Radiology Experiments (TOMCAT) scan of a xyloglucan (XG) aerogel; and (**E**) after binarization of image D. The scale bar in C is 17 mm, and in D and E is 100 μm. PEEK = Polyether ether ketone.

**Figure 2:**
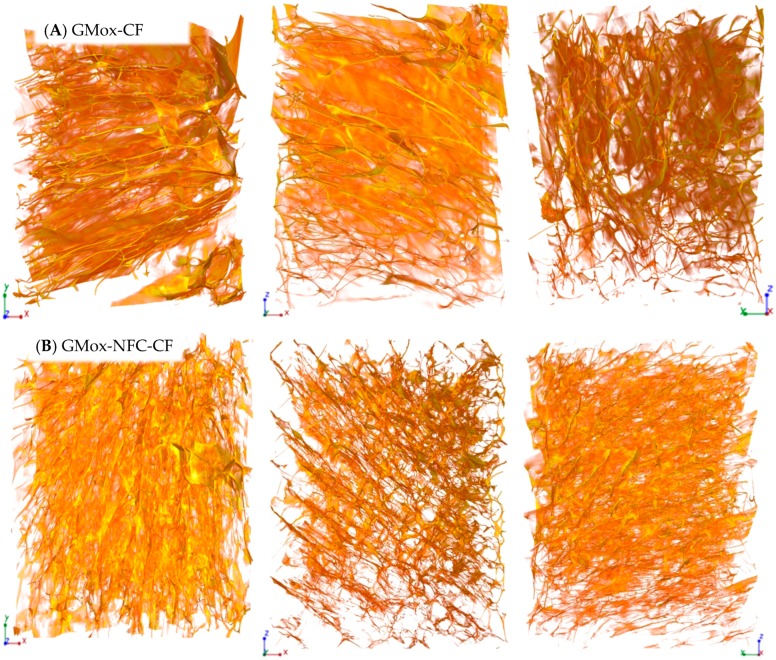
Three-dimensional reconstructed images from synchrotron phase contrast microtomography of (**A**) oxidized guar galactomannan (GM) (GMox) aerogel and (**B**) aerogel reinforced with 25% nanofibrillated cellulose (NFC) (GMox-NFC), prepared by conventional freezing (CF). The size is 495.33 × 495.33 × 660.33 μm^3^ in XYZ-axis.

**Figure 3:**
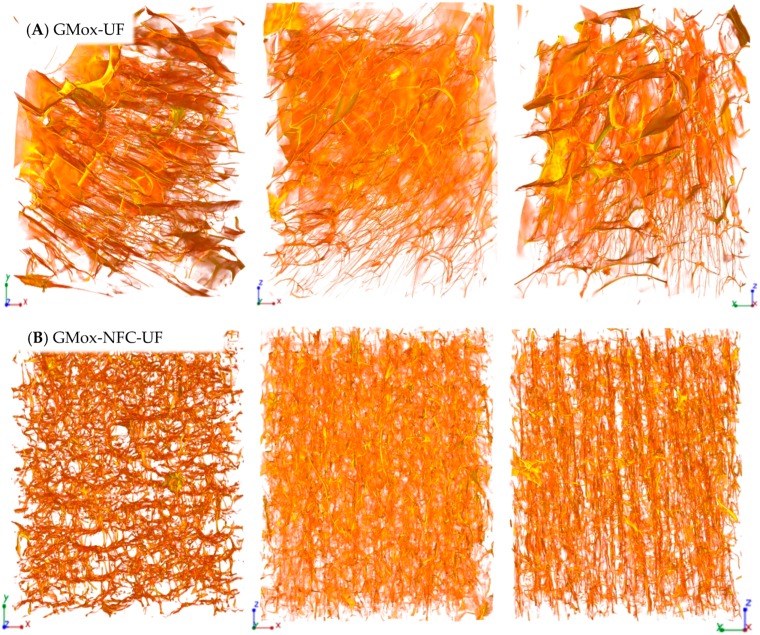
Three-dimensional reconstructed images from synchrotron phase contrast microtomography of (**A**) oxidized GM (GMox) aerogel and (**B**) aerogel reinforced with 25% NFC (GMox-NFC), prepared by unidirectional freezing (UF) using liquid nitrogen. The size is 495.33 × 495.33 × 660.33 μm^3^ in XYZ-axis.

**Figure 4:**
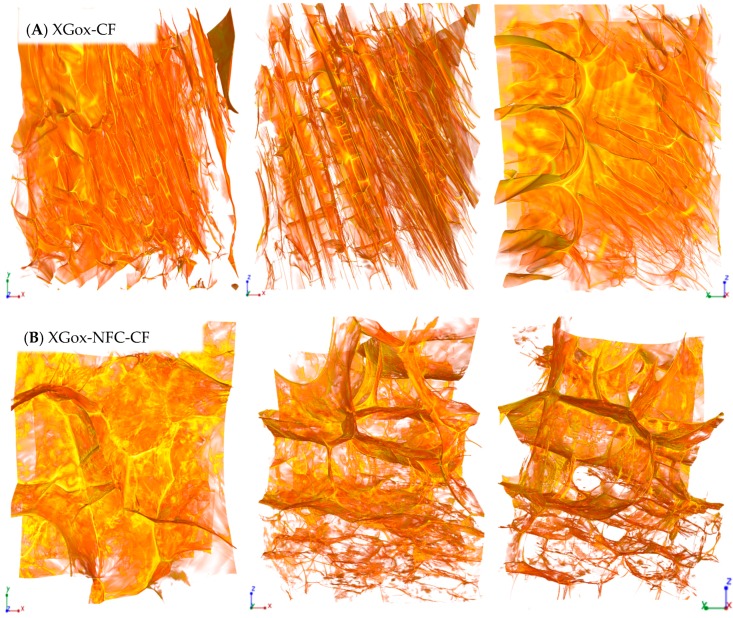
Three-dimensional reconstructed images from synchrotron phase contrast microtomography of (**A**) oxidized XG (XGox) aerogel and (**B**) aerogel reinforced with 25%NFC (XGox-NFC), prepared by conventional freezing (CF). The size is 495.33 × 495.33 × 660.33 μm^3^ in XYZ-axis.

**Figure 5:**
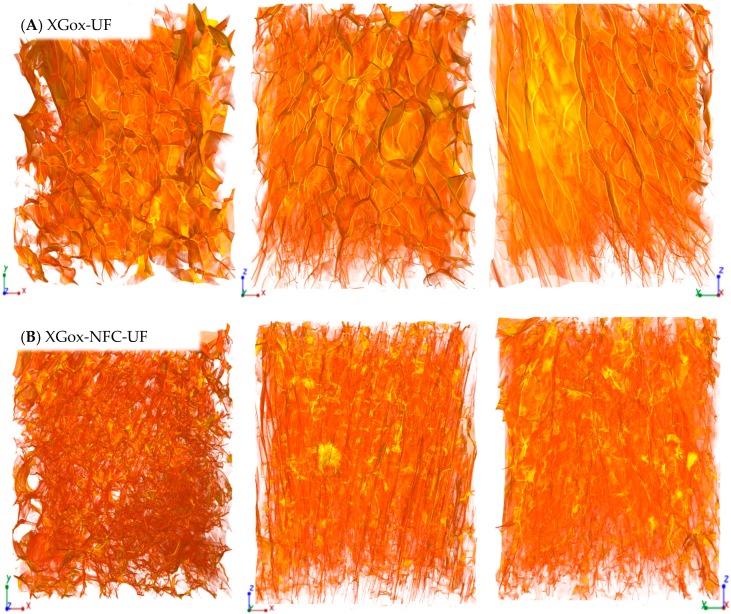
Three-dimensional reconstructed images from synchrotron phase contrast microtomography of (**A**) oxidized XG (XGox) aerogel and (**B**) aerogel reinforced with 25% NFC (XGox-NFC), prepared by unidirectional freezing (UF) using liquid nitrogen. The size is 495.33 × 495.33 × 660.33 μm^3^ in XYZ-axis.

The XMT reconstruction can be viewed as a stack of two-dimensional grayscale images (Figure 1D). These images clearly show bright areas corresponding to the polysaccharide solid structure and gray areas corresponding to the air voids. This contrast is produced due to the differences in the densities and the sample composition that directly reflect the change in the X-ray absorption in the sample. The shading (from white to gray) in the image corresponds to different X-ray absorption due to the porosities of the aerogel. 3D renderings of the reconstructed volume are presented in Figure 2, Figure 3, Figure 4 and Figure 5. The rendered volumes of all 3D images are 495.33 × 495.33 × 660.33 μm^3^. The sample codes and compositions are explained in Table 1.

GMox-CF aerogels exhibited the anisotropic structures of the pores that can easily be seen when the sample is viewed along different axes (XY-axis, XZ-axis, and YZ-axis) (Figure 2A). GMox-CF showed an intricate open cellular architecture of interconnected pores. The pore structure was irregularly arranged and showed porosities that reflected the random growth of ice crystals during conventional freezing. The pore wall consisting of a thin film that was also observed around the pores. Reinforcing the GMox aerogel with 25% NFC remarkably changed the GMox aerogel structure (Figure 2B). The pores were also anisotropic but smaller, and the structure was a highly interconnected network of polsaccharide matrix fibers in the aerogels.

When unidirectional freezing was used as an ice-templating method, the GMox-UF and GMox-NFC-UF samples showed a structure oriented in the direction of freezing (Figure 3A,B). The oriented structure as a function of the direction of the freezing was more prominent in the GMox-NFC-UF sample (Figure 3B). Irrespective of the pore size, the samples were mostly connected to form nodular channels parallel to one another in the direction of the freezing. The cell structures were anisotropic in the form of buttresses and exhibited smaller pore sizes compared to those of GMox-UF (Figure 3A). Please see the detailed 3D structure of conventional and unidirectional ice-templated GMox samples in XMT Video S1, Video S2, Video S3, and Video S4 in the supplementary data.

Conventionally frozen XGox samples exhibited notably different morphology compared to that of the GMox aerogels, in which the pore walls consisted of a well-defined thin film and the pores were heterogeneous in shape and size (Figure 4A). A thin film was also observed in the XGox-NFC-CF samples. However, the thin film was not as smooth as in the XGox-CF sample but rather was of variable thickness (Figure 4B). Hexagonal porous morphology was clearly observed in both the XGox-CF and the XGox-NFC-CF samples, where the pores were well interconnected to one another. Unidirectional freezing resulted in the smaller pore size of XGox-UF (Figure 5A). The porous structure was analogous to the honeycomb structure, and the pores were elongated in the direction of the freezing. XGox aerogel reinforced with NFC showed a very similar geometry of pores, and unidirectional freezing resulted in an aligned structure of the pores (Figure 5B). The detailed 3D structure of all XGox samples is clearly revealed in XMT Video S5, Video S6, Video S7, and Video S8 in the supplementary data.

**Figure 6:**
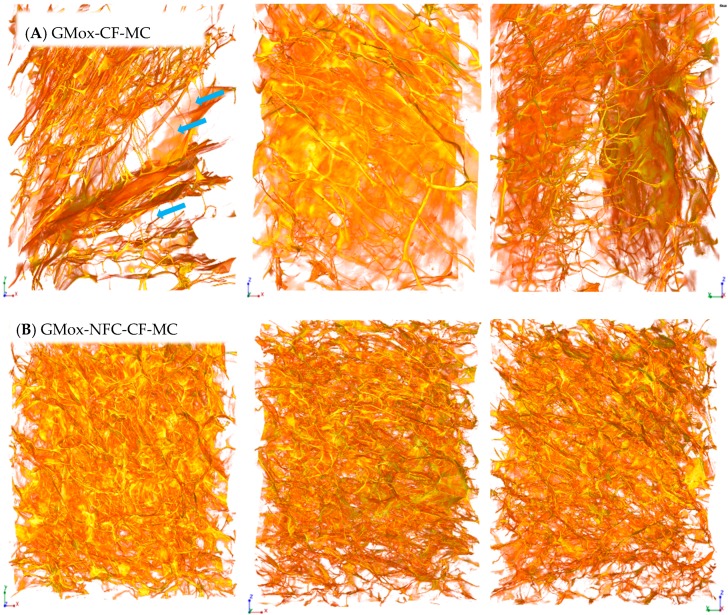
Three-dimensional reconstructed images from synchrotron phase contrast microtomography of (**A**) mechanically compressed (MC) oxidized GM (GMox-CF-MC) aerogel and (**B**) aerogel reinforced with 25% NFC (GMox-NFC-CF-MC), prepared by conventional freezing (CF). The size is 495.33 × 495.33 × 660.33 μm^3^ in XYZ-axis. Arrows indicate the observed buckling effect.

All the above mentioned aerogel samples were prepared inside capillaries (with an internal diameter of 0.8 mm). Some samples were scanned from cubical aerogels to observe the structural difference compared to the aerogels made inside a capillary. These cubical aerogels were mechanical compressed (MC), as explained in Section 3.4, and randomly carved samples (≈1 × 1 × 17 mm^3^, Length × width × height) were scanned with XMT. Figure 6 represents the morphology of the bulk material (mechanically compressed cubical aerogel). The porous structure of the GMox-CF-MC was analogous to that of the GMox-CF (Figure 2A), which means that the morphological features of the aerogels prepared in the capillaries are representative of aerogels made in cubical blocks. However, the cubical aerogel samples exhibited higher background noise, most likely due to the slight vibration of the sample during the rotation of the sample for scanning, as compared to the noise of the aerogels prepared inside a capillary. Eliminating background noise compromised the fine structure of the aerogel. The GMox-NFC-CF-MC aerogel showed a highly interconnected structure (Figure 6B) compared to that of the respective aerogel GMox-CF-MC. The aerogels obtained exhibited a flexible structure that was squeezed when a compressive force was applied. Mechanical compression did not cause structural breakdown in the aerogel; instead, it bent the structure (buckling effect) (Figure 6).

#### 2.1.1. Effect of NFC Reinforcement on the Aerogels’ Morphology

XMT imaging showed noticeable morphological differences between the plain GMox and XGox aerogels and the NFC-reinforced aerogels (Figure 2, Figure 3, Figure 4, Figure 5 and Figure 6). These 3D images elucidate the active role of NFC reinforcement on the microstructure of the matrix polysaccharide (GMox and XGox). Increasing interest in the development of biocomposites of high performance makes NFC the next-generation renewable reinforcement in the polymer matrix [33]. The catalytic reaction of the GaO enzyme was not compromised with the addition of NFC to the matrix polysaccharide solutions prior to oxidation, and oxidation entrapped NFC in the polysaccharide structure, resulting in a strong network of hydrogels [15]. That network was hypothesized to hamper the growth of ice crystals during freezing, and therefore smaller pores were formed compared to those in the plain GMox and XGox aerogels.

The purpose of adding a reinforcing agent to the composite material is to enhance the latter’s mechanical properties. The compatibility and interaction of the reinforcing agent with the polymer are imperative to achieve a high-performance composite material. To locate NFC in the GMox and XGox matrices, we used phase-enhanced XMT for scanning aerogel samples. However, visualizing NFC in the 3D structure was difficult due to the similar attenuation of X-rays by NFC and the matrix polysaccharides. Therefore, we did not distinguish NFC from the biocomposite aerogel structure. In earlier studies, Sedighi-Gilani et al. reported that XMT successfully revealed NFC in the NFC-silica hybrid aerogel because of difference in the attenuation of the X-ray energy of NFC and silica [31].

#### 2.1.2. Effect of Freezing Method on the Aerogels’ Morphology

The viscosity/gel stiffness of the polymer is, in particular, a key parameter in the ice-templating approach because it governs the critical freezing front velocity at which the polymer structure is trapped during solidification [34]. The mechanism of the formation of different pore morphologies during ice-templating is very complex [18]. The geometry of the pores varied significantly in the biocomposite aerogels when applying unidirectional freezing (Figure 3, Figure 4 and Figure 5). Both the GMox and XGox composite aerogels followed the same trend.

Unidirectional freezing aligned the aerogel’s structure in the direction of freezing and it also favored the formation of small pores due to the very low temperature of the freezing medium (−196 °C). The rapid-freezing kinetics resulted in a higher nucleation rate of ice crystals compared to their growth speed [25]. The fast-freezing kinetic leads to the formation of a vast number of small ice crystals. In our previous study, polysaccharide aerogels, prepared by unidirectional freezing using dry ice (a solid form of carbon dioxide, −78.5 °C) in an ethanol bath, showed a well-defined oriented structure but larger pores [13]. In the conventional freezing method, whereas the lower freezing temperature kinetically favored ice crystal growth, a larger pore size is characteristic of the aerogels obtained. Other factors that influenced the growth of ice crystals and the orientation of the structure are the rheological properties of the hydrogels. Enzymatic oxidation crosslinks GMox and XGox hydrogels, and the elastic behavior of hydrogels depends on the enzymatic oxidation and polysaccharide types [15]. The XGox hydrogels showed a very flexible structure compared to that of the GMox hydrogels, which were quickly repositioned between the growing ice crystals during conventional freezing (slow freezing), and the elasticity of the polysaccharide separated the ice crystals with a thin film layer. In GMox-CF aerogels, the thin film layer is thick and random because of the stiffer structure of the GMox hydrogels. Reinforcement by NFC resulted in the rigid structure of the GMox and XGox hydrogels [15] that constrained the size of growing ice crystals. This effect played a prominent role in much smaller ice crystal growth compared to that of the plain GMox and XGox aerogels (Figure 5). The nucleation of new ice crystals and the growth of existing crystals occurred concurrently. However, if the freezing kinetics are higher, as in the case of unidirectional freezing using liquid nitrogen, then the rate of ice crystal nucleation is higher compared to the ice crystal growth rate. The increase of the ice crystals is restricted to a small size by the growth of the neighboring ice crystals. The relationship between the growth and nucleation steps will determine the ice crystal size distribution [35].

### 2.2. Quantitative Analyses

The 3D-rendered images give a detailed overview of the internal morphology of the aerogels. The digital nature of the XMT data enables further quantitative analyses such as computing the pore size and wall thickness distributions.

#### 2.2.1. Volume-Weighted Pore Size Distribution

**Figure 7:**
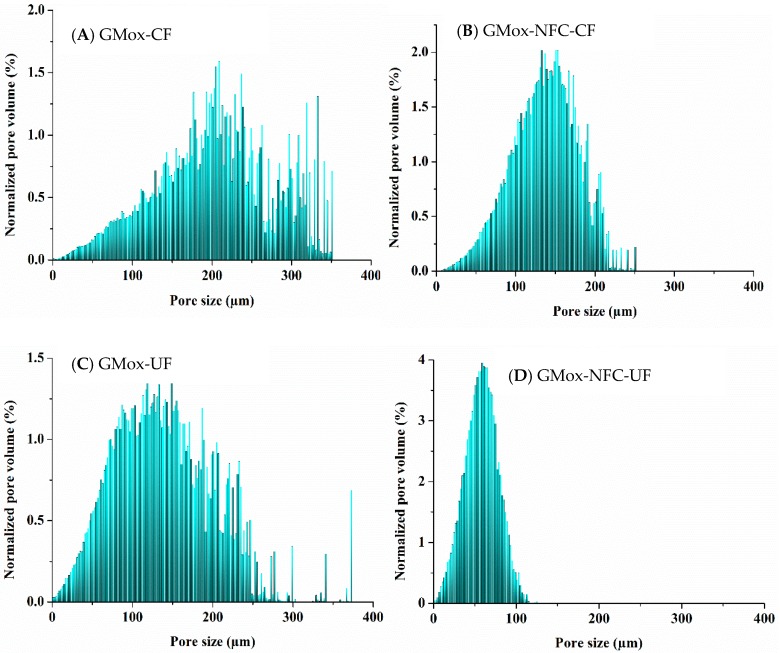
Volume-weighted pore-size distribution of (**A**) GMox and (**B**) GMox reinforced with NFC using the conventional freezing (CF) method. Volume-weighted pore-size distribution of (**C**) GMox and (**D**) GMox reinforced with NFC prepared by the unidirectional freezing (UF) method.

GMox aerogels showed a random pore size distribution; although they had some large pores of over 300 μm, most of the pores were distributed in the range of 100 μm to 250 μm (Figure 7A). The addition of NFC to GMox aerogels not only reduced the pore size but also narrowed the pore size distribution, which shows most of the pores between 100 μm and 200 μm (Figure 7B), which corroborates the effect of NFC reinforcement on the pore size distribution of the polysaccharide aerogel. Arboleda et al. also reported the NFC-reinforcement effect on the pore size of an organic composite aerogel [36]. The average pore size is in accordance with our previous studies, in which a polysaccharide aerogel’s morphology was characterized with SEM and FIB-SEM [13,15], but quantitative data analyses of XMT data give a complete picture of pore size distribution. The unidirectional freezing of GMox aerogels shifted most of the pore size distribution below 200 μm (Figure 7C). Considering both parameters at the same time resulted in a pore size reduction by 50%, and most of the pores resided in the range of 25 μm to 85 μm (Figure 7D). A segmented middle slice from the local thickness map of GMox-CF, GMox-NFC-CF, GMox-UF, and GMox-NFC-UF shows the visual appearance of the pores (Figure S1 (Supplementary data).

**Figure 8:**
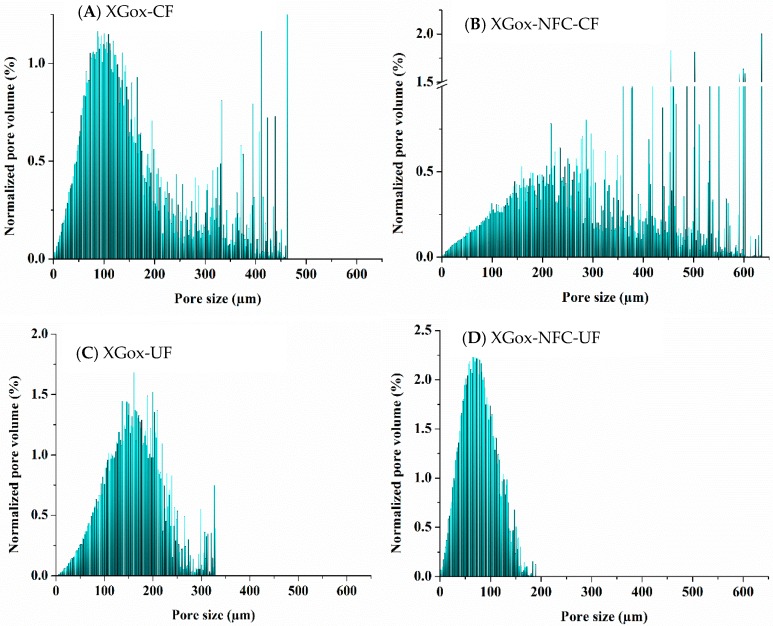
Volume-weighted pore-size distribution of (**A**) XGox and (**B**) XGox reinforced with NFC using the conventional freezing (CF) method. Volume-weighted pore-size distribution of (**C**) XGox and (**D**) XGox reinforced with NFC prepared by the unidirectional freezing (UF) method.

The XGox aerogel also exhibited pore size distribution similar to that of the GMox aerogel when prepared by the conventional freezing method. Most pores were distributed between 50 μm to 200 μm. However, some pores over 400 μm were also observed (Figure 8A). The XGox-NFC-CF aerogel showed a variation in the distribution of pore size in which almost 50% of the pore volume was in the range of 300 μm to 600 μm, even though this aerogel was reinforced with 25% NFC (Figure 8B). Since the aerogels were prepared inside a capillary with an internal diameter of 0.8 mm and there was a possibility that the volume of interest in the XMT scan did not contain a proper aerogel mass, this could be one reason for the large pore size and heterogeneity in the sample. Pores in the aerogel are heterogeneous in size and shape, and the pore walls consist of a thin film that may cause the weak absorbance of the X-rays. There is also chance that the preprocessing of data for quantitative analyses diminished the interface between the pores or that the walls were too thin to be detected at a 330 nm voxel size, which would result in some neighboring pores being treated as single large pores during local thickness calculations. The segmented middle slice from the local thickness map of XGox-CF, XGox-NFC-CF, XGox-UF, and XGox-NFC-UF shows the visual appearance of the pores (Figure S2 (Supplementary data)). The XGox-UF and XGox-NFC-UF aerogels exhibited similar pore size distributions (Figure 8C,D) as the GMox-UF and GMox-NFC-UF aerogels (Figure 7C,D). The pore-size distribution of polysaccharide aerogels can be tailored by the addition of an NFC reinforcement, and fast freezing further added the real counterpart in the narrowing of pore-size distributions.

**Figure 9:**
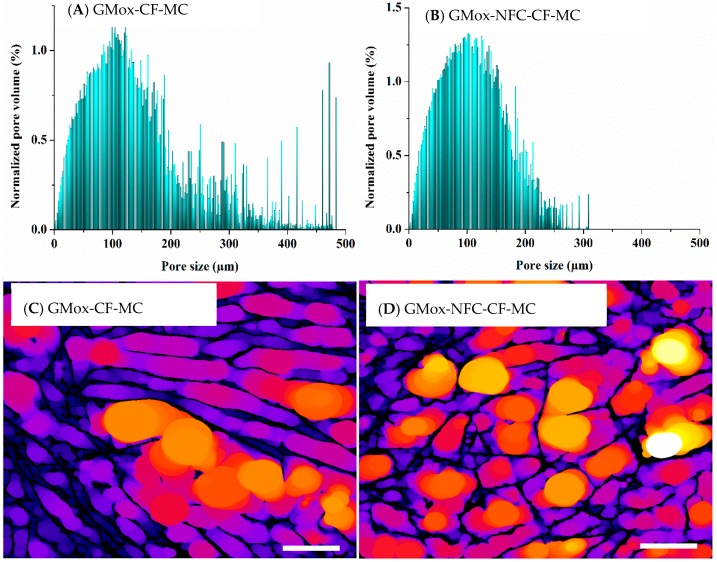
Volume-weighted pore-size distribution of (**A**) GMox and (**B**) GMox-NFC. Segmented middle slice (s1080/2160) from the local thickness map of (**C**) GMox and (**D**) GMox-NFC for pore-size distribution. Aerogels were prepared by the conventional freezing (CF) method. MC = mechanically compressed. The scale bar in C and D is 100 μm.

When comparing the pore-size distributions of the mechanically compressed cubical aerogel samples, most pores in the GMox-CF-MC were below the 200 μm mark, but some were larger than 400 μm (Figure 9A). The GMox-NFC-CF showed 50% of pores distributed below 100 μm and the remainder between 100 μm and 250 μm, with some pores of 300 μm (Figure 9B).

#### 2.2.2. Volume-Weighted Pores Wall Thickness Distribution

**Figure 10:**
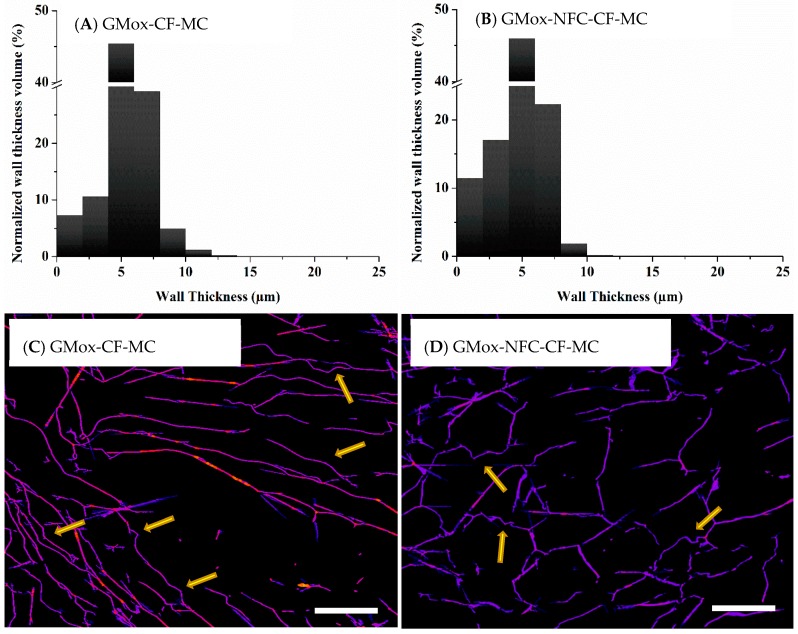
Volume-weighted pore-wall thickness distribution of (**A**) GMox and (**B**) GMox-NFC. Segmented middle slice (s1080/2160) from the local thickness map of (**C**) GMox and (**D**) GMox-NFC for pore-wall thickness distribution. Aerogels were prepared by the conventional freezing (CF) method. MC = mechanically compressed. The scale bar in C and D is 100 μm. Arrows indicate the observed buckling effect.

The pore wall thickness of GMox and GMox-NFC, regardless of the freezing method, was mostly in the range of 3 μm to 7 μm. However, a pore wall thickness of 25 μm was observed in the aerogels prepared by unidirectional freezing (Figure S3 (Supplementary data)). The same results were also true for the XGox and XGox-NFC aerogels (Figure S4 (Supplementary data)). Aerogel samples of GMox and GMox-NFC from the mechanically compressed cubical sample (Figure 10A,B) also showed pore wall thicknesses comparable to those of the aerogel samples prepared inside the capillaries.

Lyophilization also affects the external and internal structure caused by shrinkage [1]. The aerogels obtained from enzymatically oxidized GMox and XGox (plain) and their composite aerogels with NFC did not show external volumetric shrinkage. However, we observed the effect of drying on the internal structure of the aerogels. When the ice crystals sublimated, the internal structure relaxed from the stresses exerted by the ice crystals, causing the bending or buckling of the pore walls. In Figures S5 and S6 (Supplementary data), arrows point out the buckling effect. The structure of the aerogel during compression was squeezed, which resulted in a more clearly visible buckling effect (Figure 10C,D).

### 2.3. Structural Response to Uniaxial Mechanical Compression

**Figure 11:**
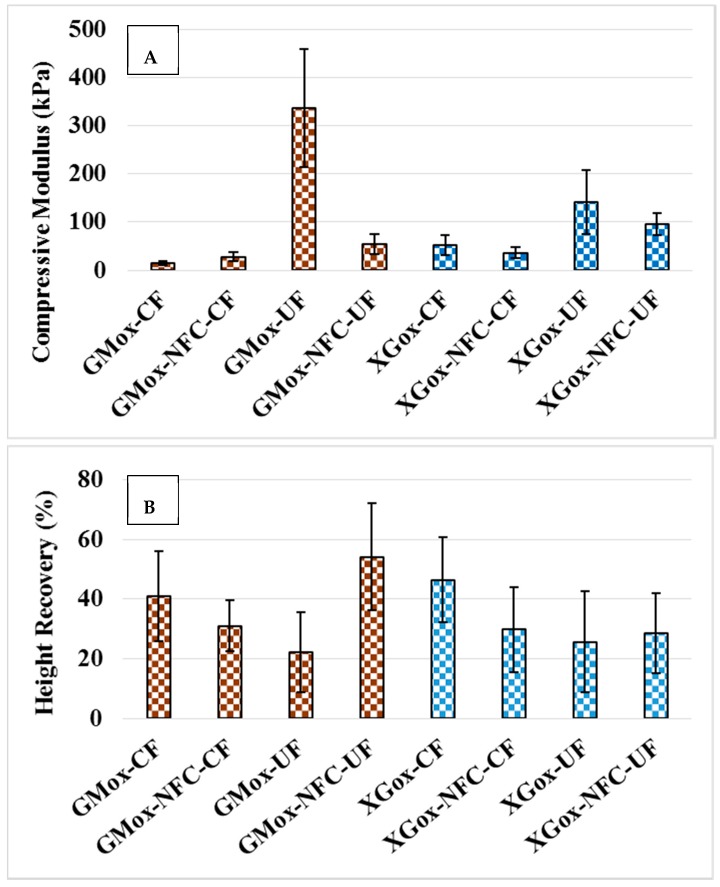
(**A**) Compressive modulus and (**B**) height recovery after the compression testing of the aerogels. GM = guar galactomannan, XG = tamarind xyloglucan, ox = oxidized, NFC = nanofibrillated cellulose, CF = conventional freezing, UF = unidirectional freezing.

The mechanical behavior and shape recovery of the porous material depend on the geometry of the pores (shape and size) in the 3D architecture of the material [25,37] and the density [14,15]. The density of all aerogels is considered the same, as the final concentration of biopolymers was kept constant (1 wt % dry content) to circumvent the effect of density on the aerogels’ morphology and mechanical behavior as well. The compressive moduli of the aerogels were in the range of 16 kPa to 330 kPa, depending on the polysaccharide types, the addition of NFC, and the ice-templating method (Figure 11A). All aerogels prepared by the unidirectional ice-templating method exhibited a higher compressive modulus than the corresponding aerogels prepared by conventional freezing. The same results have been reported in earlier studies for unidirectional ice-templated polysaccharide aerogels [13,32]. GMox-UF aerogels showed a higher compressive modulus compared to the other aerogels. Unidirectional ice-templating created a structure parallel to the applied stress, and, therefore, these aerogels showed higher resistance to compressive stress. Also, larger pore walls contributed to the higher compressive modulus of GMox-UF. The GMox-NFC-UF aerogel exhibited narrow pore size distribution (≤100 μm), and, therefore, the channels did not form in the structure. When compressive stress was applied to the aerogels, it was equally distributed throughout the structure, which resulted in a lower compressive modulus. The XGox-UF and XGox-NFC-UF aerogels also followed the same trend. Reinforcement with NFC (25%) clearly exhibited significant effects on narrowing the pore size distributions. However, the effect of NFC on the mechanical properties was not clearly observed. In our earlier studies, NFC showed clearly positive effects on the mechanical behavior of conventionally frozen GMox and XGox aerogels and those prepared by supercritical CO_2_ drying [14,15]. However, the mechanical properties of ice-crystal templated, NFC-reinforced aerogels from GMox and XGox were studied for the first time in the present paper. GMox-NFC-CF aerogels displayed a higher compressive modulus compared to GMox-CF. However, XGox-NFC-CF aerogels did not follow this trend, and the same was also true for both GMox-NFC-UF and XGox-NFC-UF aerogels. The results indicate that the reinforcing effect of NFC depends on the source and processing technology of NFC. In the present work, native NFC was used, which was obtained by only mechanical disintegration, having a larger fibril structure (10 nm to 50 nm wide and several micrometers long) compared to previously studied types of NFC [14,15]. This plain NFC sample behaved as a liquid-like suspension at a similar dry matter content as that at which anionic NFC (Fiber width of 4–10 nm, a length of several micrometers, and chemically modified with anionic groups), that was studied earlier, was a gel [14]. Thus, the gel forming ability and most probably the reinforcing capacity of this NFC was not comparable to other types of NFC used in our earlier work.

The shape recovery (height recovery after compression) of the aerogels is presented in Figure 11B. The GMox-UF and GMox-NFC-UF aerogels exhibited shape recovery contrary to that of the corresponding aerogels prepared by conventional freezing. Shape recovery of the GMox and XGox aerogels was higher than that of the corresponding aerogels containing NFC as a reinforcement when the conventional freezing method was used. Two possible reasons could explain the greater shape recovery: first, the addition of NFC to GMox and XGox aerogels decreased the distribution of pore size, and, second, NFC reinforcement made the pore walls stiffer and less elastic. An earlier study, in which composite aerogels were prepared from *O*-acetylgalactoglucomannans (GGM) and NFC crosslinked with ammonium zirconium carbonate, showed that aerogels with a higher NFC content exhibited less shape recovery. A negative correlation was observed between compression shape recovery and the NFC content and compressive modulus. However, NFC showed positive effects on the GGM and NFC composite aerogels [38]. During compression, the large pore walls were folded perpendicularly to the applied stress, and the small pore walls within the large pore walls maintained their shape. The large pore walls parallel to the applied stress buckled. The unfolding of and the recovery from the buckled structure resulted in higher shape recovery, but only if the permanent deformation of pore walls did not occur. The larger pore walls of the GMox-UF buckled to their maximum capacity, and, beyond that point, compressive stress caused permanent deformation in the aerogel structure (Figure S7, Supplementary data). This led to less shape recovery (Figure 11B). The GMox-NFC-UF aerogels showed little permanent deformation (Figure S7, Supplementary data) and structurally bounced back to a higher shape recovery after releasing the stress (Figure 11B). The shape recovery and compressive moduli of the XGox and XGox-NFC aerogels also followed the same trend in the freezing method (Figure 11B). The addition of NFC narrowed the pore-size distributions and made the pore walls stiffer, which quickly dissipated the stress applied throughout the structure, and, hence, the bouncing back of the aerogel structure decreased. This phenomenon was explained earlier by Sauter et al. [37] for polyether urethane foam. During compression testing, we also heard sounds, especially from the XGox-UF and XGox-NFC-UF samples, that probably occurred due to the structure’s breakdown.

The visual shape deformation of the cubical aerogel samples after compression testing is presented in Figure S7 (Supplementary data). The aerogel samples prepared by unidirectional freezing showed an uneven deformation of the cubic shape. Unidirectional freezing caused the formation of columns by aligning the structure in the direction of freezing. During compression testing, these structural columns were bent from the weak point, exhibiting a buckling effect. If the compressive stress is less than the stress required for permanent buckling, the material can recover its maximum shape when the applied stress is released. GMox-NFC-UF showed a higher shape recovery (Figure 11B and Figure S7 (Supplementary data)). The buckling effect was observed less in the aerogel samples prepared by conventional freezing compared to those prepared by unidirectional freezing. The deformation of the shape of the aerogel cube was not uniform, particularly in the samples prepared by unidirectional freezing.

## 3. Materials and Methods

### 3.1. Materials

Guar galactomannan (GM) obtained from Sigma-Aldrich (St. Louis, MO, USA) and tamarind seed xyloglucan (XG), a gift from Dainippon Sumitomo Pharma, Osaka, Japan, were used as aerogel-forming materials. NFC refined from birch pulp without chemical derivatization was purchased from UPM, Finland, and was used as a reinforcing agent. The NFC fiber was 10 nm to 50 nm wide and several micrometers long. The galactose oxidase (GaO) used was from *Fusarium* spp., which was produced recombinantly in *Picihia pastoris* and donated by Dr. Sybe Hartmans (DSM Biotechnology Center, The Netherlands). Horseradish peroxidase (HRP, P8250, Type II, 181 U/mg) and catalase (C30, from bovine liver, 22,000 U/mg) were obtained from Sigma-Aldrich (St. Louis, MO, USA).

### 3.2. Enzymatic Crosslinking and Aerogel Formation

GM and XG hydrogels were prepared using the previously explained method [13,15]. NFC gel (1 wt % dry content) was added to the GM and XG solutions before enzymatic oxidation at 25 wt % of the total weight of the polysaccharides (GM/XG). The enzyme dosages (GaO: 1.8–4.5 U/mg of galactose, catalase: 115 U/mg, HRP: 1.5 U/mg) were based on previous studies [12,15]. After the enzymatic treatment, oxidized GM (GMox)- and XG (XGox)-based hydrogels were obtained, and the enzymes were inactivated by heating the samples in boiling water for 5 min. 

For synchrotron radiation microtomography, the enzyme-inactivated hydrogels, from which air bubbles were removed by vacuum, were injected into polyether-ether-ketone (PEEK) capillaries (wall thickness = 0.0175 mm and internal diameter = 0.8 mm) using a small syringe (Figure 1A,B) and placed vertically into hydrogels cast in cubical molds. The cubical aerogels (GMox and XGox) were also prepared without the PEEK capillaries (Figure 1C). The hydrogels were frozen using conventional freezing at −70 °C for 3 h to 4 h. For unidirectional freezing, a cubical petri dish was placed on a liquid nitrogen surface (≈20 min) separated by aluminum foil (three to four layers) to avoid the direct contact of the petri dish with the liquid nitrogen. In unidirectional freezing, the hydrogels were frozen from the bottom toward the z-direction (Figure 1B). Lyophilization of the pre-frozen hydrogels was performed at 1 mbar atmospheric pressure for about 48 h to obtain aerogels. The term ‘polysaccharide’ refers to only matrix polysaccharide (GMox/XGox) in this paper. The sample codes and compositions are explained in Table 1.

### 3.3. Synchrotron X-rays Tomography (XMT) Scanning and Image Reconstruction

Aerogel samples in the PEEK capillaries were glued on carbon fiber rods (diameter = 3 mm and length = 10 mm). All the samples were scanned at a height of 6 mm from the bottom to avoid any artifacts from the glue. Also, small samples (≈ 1 × 1 × 17 mm^3^ in length, width, and height) from mechanically compressed (see Section 3.4) cubical aerogel samples were carved with a razor blade and glued onto the carbon rods. We conducted XMT scans on the oxidized GM (GMox) and oxidized XG (XGox) aerogels with and without NFC reinforcement (25%) at the beamline for TOmographic Microscopy and Coherent rAdiology experimenTs (TOMCAT) at the Paul Scherrer Institut (PSI) in Villigen, Switzerland. The data were acquired with an X-ray energy of 17.5 keV. After the penetration of the sample, X-rays were converted into visible light by a LuAG:Ce scintillator screen (20 μm, Crytur, Turnov, Czech Republic). The visible light was magnified using ×20 magnification, diffraction-limited microscope optics and recorded with a 2560 × 2160 pixel sCMOS camera (pco.edge 5.5, PCO, Kelheim, Germany). Thus, the voxel side length was 0.33 μm, and the exposure time for each of the 1501 projections was 150 ms. The projections were reconstructed using the single-distance phase retrieval Paganin algorithm [39], in which the delta and beta values were chosen in such a way as to avoid excessive blurring while increasing the contrast in the images in order to increase the contrast of the aerogel matrix in the reconstructions. Details of the TOMCAT reconstruction pipeline are described in [40,41]. Parts of the PEEK capillaries were removed by selecting the region of interest (ROI) for the aerogel matrix before data analysis, in which the z-direction showed the stack of images (Figure 1B). In the data, the z-direction showed the stack of images. The VGStudioMax 2.1 software (Volume Graphics GmbH, Heidelberg, Germany) was used for 3D visualizations of the aerogel structures.

Quantitative data analyses such as volume-weighted pore-size distribution and volume-weighted wall thickness distribution were calculated using the Local Thickness plugin to ImageJ software (http://imagej.net/Local_Thickness) [42]. In this method, the pore size for each voxel in the dataset was treated as ‘the diameter of the largest sphere that fits inside the pore space and contains the voxel’, and the wall thickness defined for each voxel as ‘diameter of the largest sphere that fits inside the aerogel material and contains the voxel’ [42]. The local thickness plugin minimizes the effect of aliasing artifacts on the results because it does not require separating each pore. For quantitative analysis, first, a bilateral filter was applied to reduce noise and smooth the images. The image was binarized (converted from grayscale (Figure 1D) to a black-and-white image) using a dual threshold (hysteresis) segmentation (Figure 1E). After measuring the local thickness map of the pores and pore walls, the histograms of the two data sets were calculated and presented as the volume-weighted pore size and wall thickness distributions. All the quantitative data analyses have been performed using the facilities at the Center for Scientific Computing (CSC—IT Center for Science Ltd., Espoo, Finland).

### 3.4. Mechanical Testing

The Instron 33R4465 universal testing machine (Instron Corp., High Wycombe, UK) with a load cell of 100 N was used for the mechanical testing of aerogels. Cubical aerogel samples (17 × 17 × 17 mm^3^) were compressed vertically for 6 mm at a rate of 1.3 mm/min. The unidirectional frozen aerogel samples were compressed in the freezing direction (vertical direction). The compressive modulus was determined from the stress-strain curve of the aerogels. Also, the sample recovery after compression was calculated by subtracting the height of the compressed sample from that of the uncompressed sample. The obtained value was then subtracted from the compression height (6 mm) to get the recovered height of the sample. Before the measurements, the aerogel samples were conditioned for 48 h in desiccators containing Mg(NO_3_)_2_ to set the relative humidity between 50% and 55%. All measurements were performed at 21 °C and 50% relative humidity (RH). The data were obtained from 10 replicates of each aerogel sample.

## 4. Conclusions

The microstructure of the polysaccharide-based aerogels was successfully visualized in three-dimensions using the phase-retrieval synchrotron XMT. The morphological analysis performed in small samples in the capillaries provided a representative illustration of the bulky cubical aerogels, indicating that XMT can be conveniently used for the characterization of aerogels. The digital nature of the data enabled quantitative analyses such as the calculation of the pore-size and pore-wall-thickness distributions. Ice-templating methods showed a high impact on the pore-size distribution. Unidirectional ice-templating using liquid nitrogen resulted in a narrow and more homogenous pore-size distribution compared to what happened with the conventional ice-templating method, regardless of the matrix polysaccharide type (GMox/XGox). NFC reinforcement exhibited an indubitable effect on the pore-size distributions, and this effect was more prominent when the unidirectional ice-templating method was used. NFC reinforcement also played its role in the rheological properties of the matrix polysaccharide (GMox/XGox) hydrogels. Pore-wall-thickness distributions were more or less independent of the processing conditions and the reinforcement agent. However, the mechanical properties such as shape recovery and compressive modulus were dependent on the pore-size distributions, which is the outcome of the nucleation and ice crystal growth kinetics of the ice-templating methods. This study provided very useful practical information that helps in the design of microporous materials of a desired morphology. Carefully controlling the nucleation and ice crystal growth results in the optimal properties of the porous material.

## Figures and Tables

**Table 1:** Samples compositions and processing conditions.

Samples Codes *	Amount of NFC (wt %)	Ice-Templateing Method *
**GMox-CF**	0	CF
**GMox-NFC-CF**	25	CF
**GMox-UF**	0	UF
**GMox-NFC-UF**	25	UF
**XGox-CF**	0	CF
**XGox-NFC-CF**	25	CF
**XGox-UF**	0	UF
**XGox-NFC-UF**	25	UF
**GMox-CF-MC**	0	CF
**GMox-NFC-CF-MC**	25	CF

* GM = guar galactomannan, XG = tamarind seed xyloglucan, ox = enzymatically oxidized, NFC = nanofibrillated cellulose, CF = conventional freezing, UF = unidirectional freezing. MC = mechanically compressed.

## Supplementary Materials

The following are available online at www.mdpi.com/1996-1944/10/8/871/s1. Video S1: Video (360° rotation) of 3D reconstructed structure of oxidized GM (GMox) aerogel prepared by conventional freezing (CF), Video S2: Video (360° rotation) of 3D reconstructed structure of oxidized GM (GMox) aerogel reinforced with 25% NFC (GMox-NFC), prepared by conventional freezing (CF), Video S3: Video (360° rotation) of 3D reconstructed of oxidized GM (GMox) aerogel prepared by unidirectional freezing (UF) using liquid nitrogen, Video S4: Video (360° rotation) of 3D reconstructed structure of oxidized GM (GMox) aerogel reinforced with 25% NFC (GMox-NFC), prepared by unidirectional freezing (UF) using liquid nitrogen, Video S5: Video (360° rotation) of 3D reconstructed structure of oxidized XG (XGox) aerogel prepared by conventional freezing (CF), Video S6: Video (360° rotation) of 3D reconstructed structure of oxidized XG (XGox) aerogel reinforced with 25% NFC (XGox-NFC), prepared by conventional freezing (CF), Video S7: Video (360° rotation) of 3D reconstructed structure of oxidized XG (XGox) aerogel prepared by unidirectional freezing (UF) using liquid nitrogen, Video S8: Video (360° rotation) of 3D reconstructed structure of oxidized XG (XGox) aerogel reinforced with 25% NFC (XGox-NFC), prepared by unidirectional freezing (UF) using liquid nitrogen. Figure S1: Segmented middle slice (s1080/2160) from Local thickness map of GMox (A) and GMox-NFC (B) prepared by conventional freezing (CF) method and unidirectional freezing (UF) method (C & D), respectively, for pore size distribution. Figure S2: Segmented middle slice (s1080/2160) from Local thickness map of XGox (A) and XGox-NFC (B) prepared by conventional freezing (CF) method and unidirectional freezing (UF) method (C & D), respectively, for pore size distribution. Figure S3: Volume weighted pore wall thickness distribution of GMox (A) and GMox reinforced with NFC (B) using conventional freezing (CF) method. Volume weighted pore wall thickness distribution of GMox (C) and GMox reinforced with NFC (D) prepared by unidirectional freezing (UF) method. Figure S4: Volume weighted pore wall thickness distribution of XGox (A) and XGox reinforced with NFC (B) using conventional freezing (CF) method. Volume weighted pore wall thickness distribution of XGox (C) and XGox reinforced with NFC (D) prepared by unidirectional freezing (UF) method. Figure S5: Segmented middle slice (s1080/2160) from Local thickness map of GMox (A) and GMox-NFC (B) prepared by conventional freezing (CF) method and unidirectional freezing (UF) method (C & D), respectively, for pore wall thickness distribution. Arrows indicate the observed buckling effect. Figure S6: Segmented middle slice (s1080/2160) from Local thickness map of XGox (A) and XGox-NFC (B) prepared by conventional freezing (CF) method and unidirectional freezing (UF) method (C & D), respectively, for pore wall thickness distribution. Arrows indicate the observed buckling effect. Figure S7: Cubical samples for aerogels after mechanical compression test. GM = guar galactomannan, XG = tamarind xyloglucan, ox = oxidized, NFC = nanofibrillated cellulose, CF = conventional freezing, UF = unidirectional freezing.

Supplementary File 1

Supplementary File 2

Supplementary File 3
