# Supplementary material for: Synchrotron Microtomography Reveals the Fine Three-Dimensional Porosity of Composite Polysaccharide Aerogels
Source: Materials (Basel). 2017 Jul 28;10(8):871. doi: 10.3390/ma10080871 (PMC5578237; doi:10.3390/ma10080871)
Supplement: Supplementary File 1 [file materials-10-00871-s001.pdf]

Supplementary Materials: Synchrotron Microtomography Reveals the Fine Three-Dimensional Porosity of Composite Polysaccharide Aerogels

Abdul Ghafar, Kirsti Parikka, David Haberthür, Maija Tenkanen, Kirsi S. Mikkonen, and Jussi-Petteri Suuronen

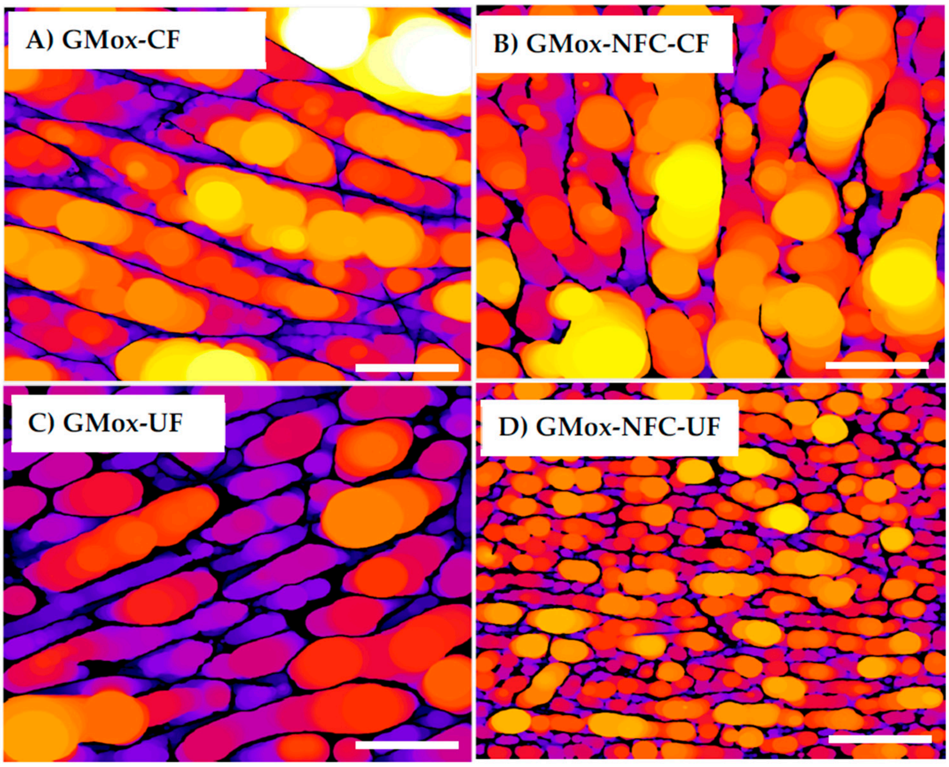

Figure S1: Segmented middle slice (s1080/2160) from local thickness map of (A) GMox and (B) GMox-NFC prepared by (C and D) the conventional freezing (CF) method and the unidirectional freezing (UF) method, respectively, for pore size distribution. Scale bar is 100 μm.

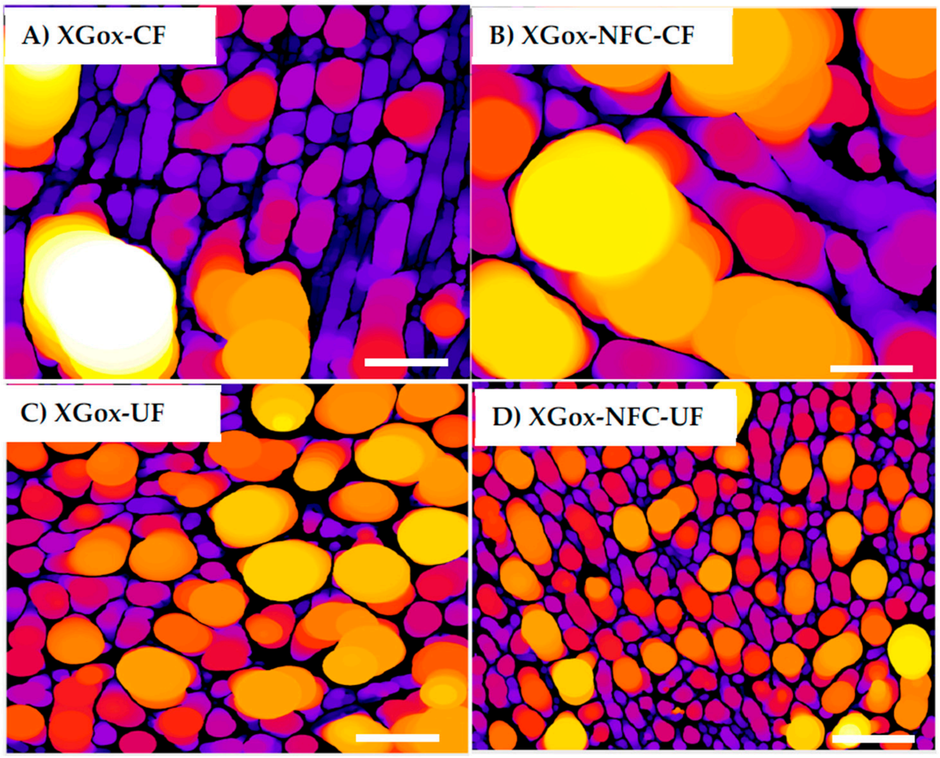

Figure S2: Segmented middle slice (s1080/2160) from local thickness map of (A) XGox and (B) XGox-NFC prepared by (C and D) the conventional freezing (CF) method and the unidirectional freezing (UF) method, respectively, for pore size distribution. Scale bar is 100 µm.

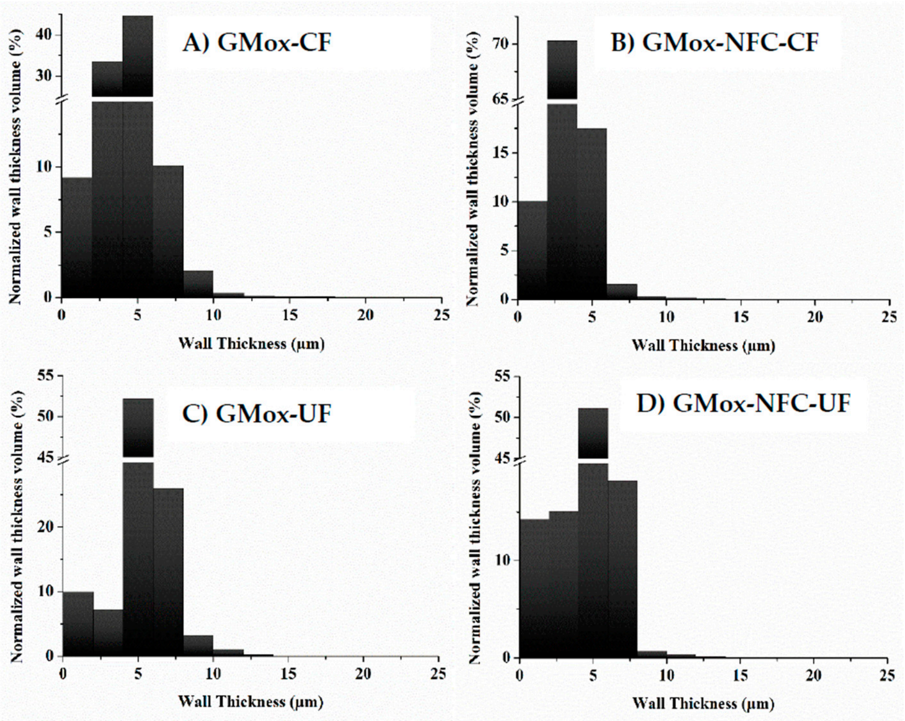

Figure S3: Volume weighted pore wall thickness distribution of (A) GMox and (B) GMox reinforced with NFC using the conventional freezing (CF) method. Volume weighted pore wall thickness distribution of (C) GMox and (D) GMox reinforced with NFC prepared by the unidirectional freezing (UF) method.

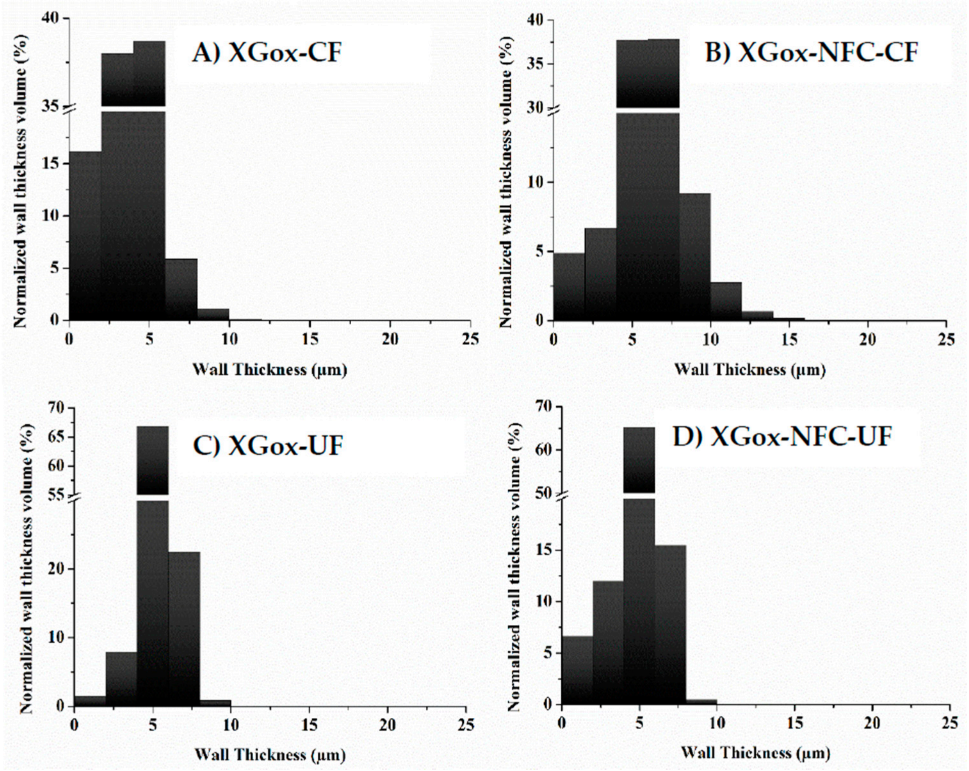

Figure S4: Volume weighted pore wall thickness distribution of (A) XGox and (B) XGox reinforced with NFC using the conventional freezing (CF) method. Volume weighted pore wall thickness distribution of (C) XGox and (D) XGox reinforced with NFC prepared by the unidirectional freezing (UF) method.

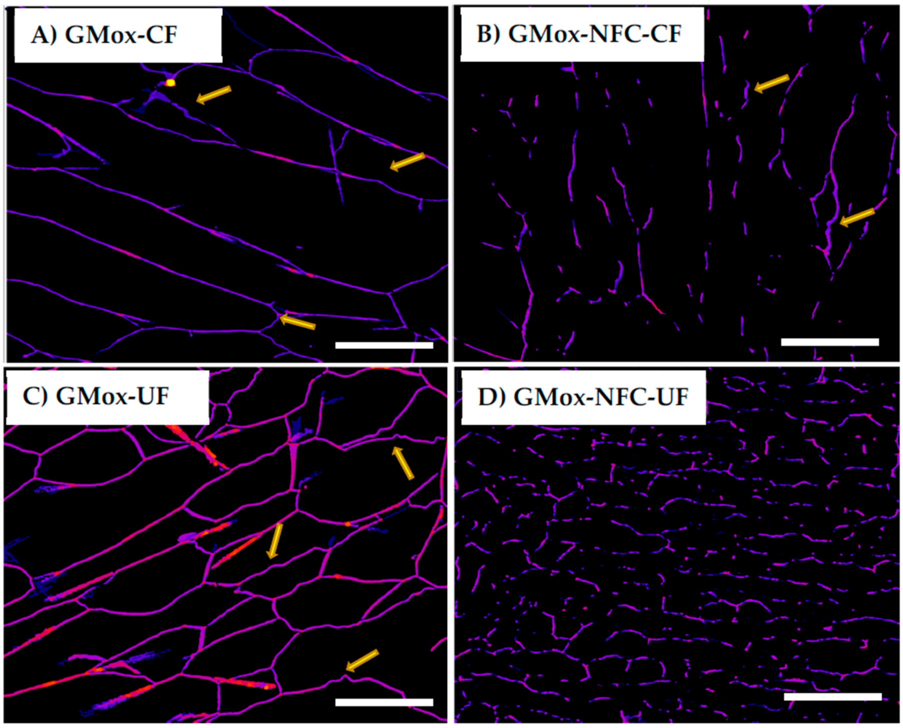

Figure S5: Segmented middle slice (s1080/2160) from local thickness map of (A) GMox and (B) GMox-NFC prepared by (C and D) the conventional freezing (CF) method and unidirectional freezing (UF) method, respectively, for pore wall thickness distribution. Scale bar is 100 µm. Arrows indicate the observed buckling effect.

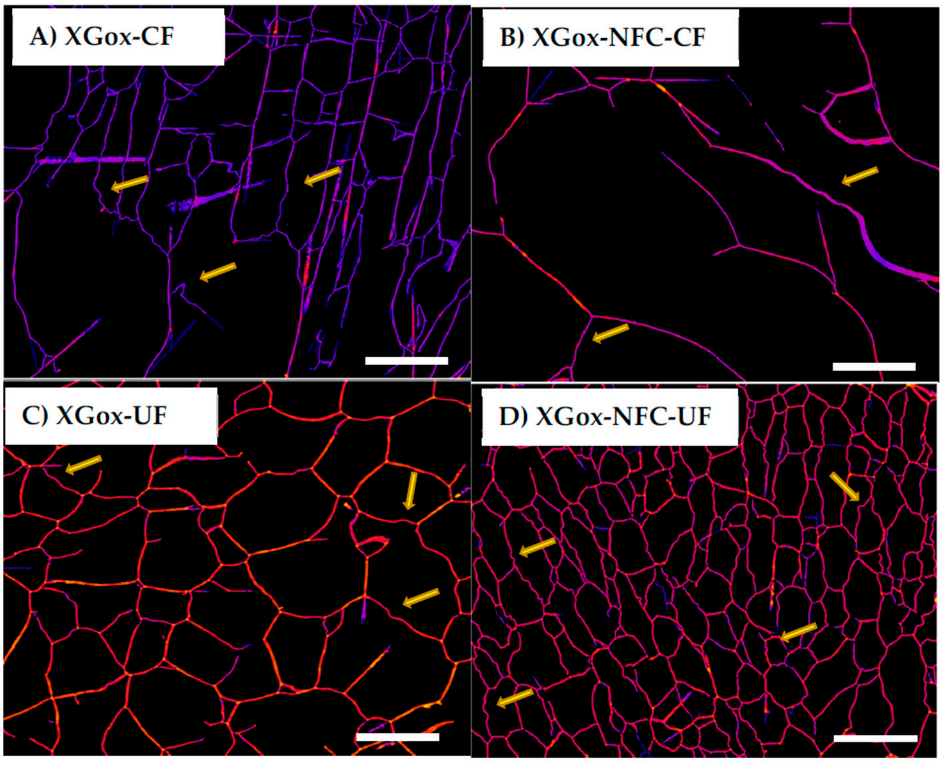

Figure S6: Segmented middle slice (s1080/2160) from local thickness map of (A) XGox and (B) XGox-NFC prepared by (C and D) the conventional freezing (CF) method and the unidirectional freezing (UF) method, respectively, for pore wall thickness distribution. Arrows indicate the observed buckling effect.

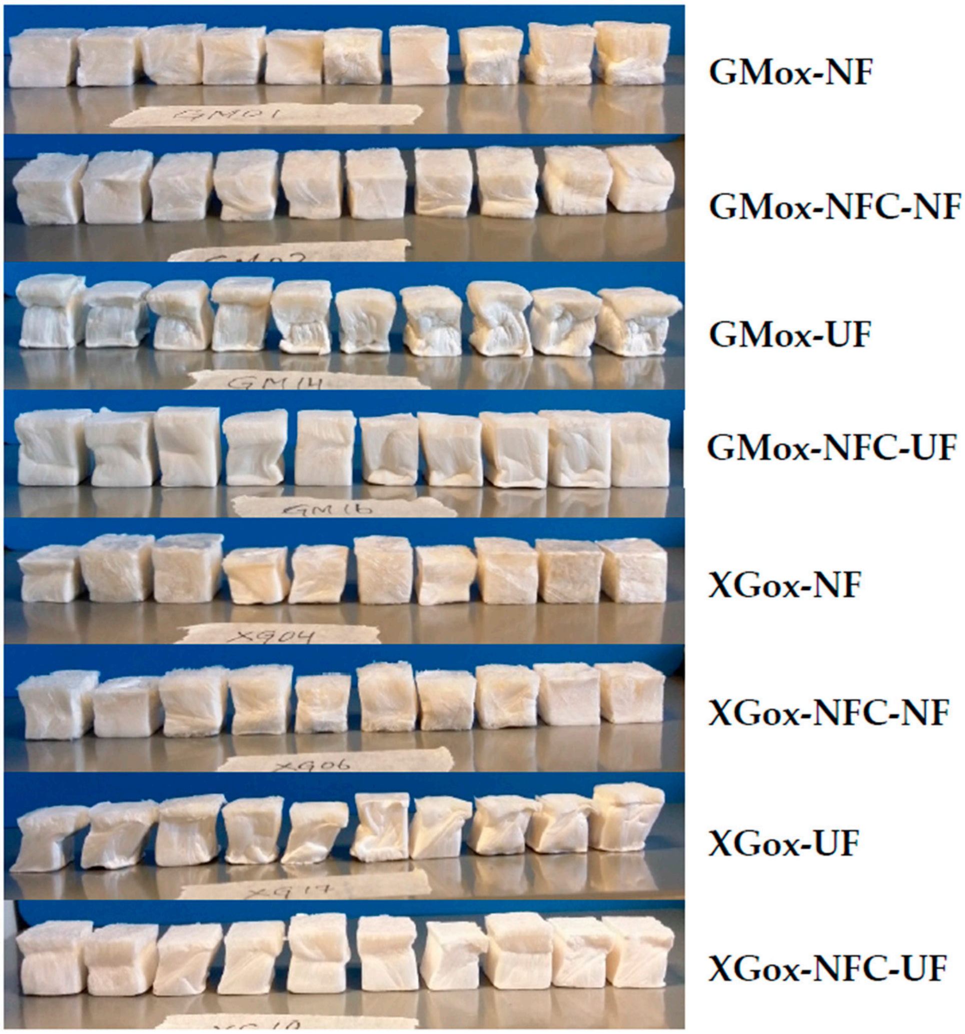

Figure S7: Cubical samples for aerogels after the mechanical compression test. GM= guar galactomannan, XG = tamarind xyloglucan, ox=oxidized, NFC = nanofibrillated cellulose, CF = conventional freezing, UF = unidirectional freezing.

© 2017 by the authors. Submitted for possible open access publication under the terms and conditions of the Creative Commons Attribution (CC BY) license (http://creativecommons.org/licenses/by/4.0/).
